# Sitagliptin monotherapy has better effect on insulinogenic index than glimepiride monotherapy in Japanese patients with type 2 diabetes mellitus: a 52-week, multicenter, parallel-group randomized controlled trial

**DOI:** 10.1186/s13098-016-0131-y

**Published:** 2016-02-27

**Authors:** Yaeko Kondo, Norio Harada, Akihiro Hamasaki, Shizuka Kaneko, Koichiro Yasuda, Eiichi Ogawa, Shin-ichi Harashima, Hiroko Yoneda, Yoshihito Fujita, Norikazu Kitano, Yoshio Nakamura, Fujio Matsuo, Megumi Shinji, Shiro Hinotsu, Takeo Nakayama, Nobuya Inagaki, The MAIKO Study group

**Affiliations:** Department of Diabetes, Endocrinology and Nutrition, Graduate School of Medicine, Kyoto University, 54 Kawahara-cho, Shogoin, Sakyo-ku, Kyoto, 606-8507 Japan; Department of Internal Medicine, Kyoto Kizugawa Hospital, Joyo, Japan; Nagisa Clinic, Hirakata, Japan; Division of Diabetes/Endocrinology/Lifestyle-related Disease, Takatsuki Red Cross Hospital, Takatsuki, Japan; Department of Diabetology and Endocrinology, Osaka Saiseikai Noe Hospital, Osaka, Japan; Department of Diabetes and Endocrinology, Shiga Medical Center for Adults, Moriyama, Japan; Department of Diabetes Mellitus, The Japan Baptist Medical Foundation, The Japan Baptist Hospital, Kyoto, Japan; Nishio Clinic, Uji, Japan; Department of Internal Medicine, Hyogo Prefectural Tsukaguchi Hospital, Amagasaki, Japan; Division of Diabetes and Endocrinology, Department of Internal Medicine, Hyogo Prefectural Amagasaki Hospital, Amagasaki, Japan; Statcom Company Limited, Bunkyo-Ku, Japan; Center for Innovative Clinical Medicine, Okayama University Hospital, Okayama, Japan; Department of Health Informatics, School of Public Health, Graduate School of Medicine, Kyoto University, Kyoto, Japan

**Keywords:** Clinical trial, Type 2 diabetes, Insulin secretion, DPP-4 inhibitor, Sulphonylurea

## Abstract

**Background:**

The 52-week monotherapy with the dipeptidyl peptidase-4 inhibitor sitagliptin and the sulphonylurea glimepiride on early-phase insulin secretion in Japanese patients with type 2 diabetes mellitus (T2DM) is not known.

**Methods:**

A randomized, parallel-group, open-label trial was conducted at 18 centers between February, 2011 and March, 2013. 171 outpatients with T2DM were recruited and randomly assigned to glimepiride or sitagliptin by minimization. Doses of glimepiride (0.25–1.0 mg/day) and sitagliptin (25–100 mg/day) were adjusted for hemoglobin A1c (HbA1c) > 6.9 %. Analyses were performed on full analysis set (FAS) of randomized subjects taking medications as allocated, and underwent 75 g oral glucose tolerance test (OGTTs) before and after treatment. The primary outcome was insulinogenic index to quantify early-phase insulin secretion after treatment, which was evaluated by analysis of covariance (ANCOVA).

**Results:**

Of 171 enrolled subjects, 68 in the sitagliptin group and 65 in the glimepiride group were included in the FAS (mean age, 64 years; baseline (HbA1c), 7.4 %). The primary outcome revealed a significantly higher insulinogenic index in the sitagliptin group than that in the glimepiride group (p = 0.036). Sitagliptin also reduced plasma glucose levels at 60 and 120 min during OGTT compared with glimepiride, while achieving a similar improvement in HbA1c during treatment. Body weight did not change in either of the two groups, and one case of hypoglycemia was observed in the glimepiride group.

**Conclusions:**

Sitagliptin shows better effects on insulinogenic index after 52-week treatment compared with glimepiride in Japanese patients with T2DM.

*Trial registration* University hospital Medical Information Network (UMIN) Clinical Trials Registry, No.00004791.

**Electronic supplementary material:**

The online version of this article (doi:10.1186/s13098-016-0131-y) contains supplementary material, which is available to authorized users.

## Background

The high prevalence of type 2 diabetes mellitus (T2DM) is a worldwide public health concern. Asian countries are currently facing a greater burden of T2DM; more than 60 % of the world’s T2DM patients are in Asia [1].

Efficacy evaluation of treatment with diabetes commonly uses hemoglobin A1c (HbA1c); however, the level of this index does not allow for evaluation of treatment effects on insulin secretion or insulin resistance [2–4]. β-cell function in patients with T2DM is approximately 50 % that of healthy individuals at the time of diagnosis, and decreases yearly thereafter [5]. Hence, evaluation of β-cell function as well as HbA1c is desirable for assessment of drug efficacy in treatment of diabetes. An important characteristic of T2DM is elevation of the fasting glucose level as well as that of postprandial glucose level, which are mainly affected by postprandial insulin secretion. Insulinogenic index is commonly used to assess early-phase insulin secretion in response to glucose [6–8]. It is reported that a reduced insulinogenic index represents the main abnormality in the transition from normal glucose tolerance (NGT) to T2DM, resulting in the elevation of postprandial glucose levels in Asian subjects [7–12]. In addition, maintenance of an appropriate insulinogenic index decreases incidence of microalbuminuria in T2DM [13]. Considered together, these findings suggest that the insulinogenic index may be a critical factor in progression to T2DM, maintenance of postprandial glucose levels, and prevention of the complications of diabetes.

The American Diabetes Association and European Association for the Study of Diabetes consensus for treating T2DM recommend biguanides as first-line therapy [14]. This treatment, however, has not been established in Asian countries, including Japan [15, 16]. Unlike the insulin resistance seen in Caucasians, Asian patients with T2DM have a relatively low BMI and a predominant insulin secretory defect [3, 7–12, 17–23]. Therefore, insulin secretagogues, particularly sulfonylureas and dipeptidyl peptidase-4 (DPP-4) inhibitors are widely used in Japan [24]. Meta-analysis has shown that DPP-4 inhibitors are more effective in Asian compared to non-Asian patients [23]. Effectiveness of DPP-4 inhibitors on insulin secretion stimulated by glucose for 12-week has shown in Korean patients with T2DM [25]. However, little is known the effects of DPP-4 inhibitors on the insulinogenic index as the primary endpoint compared to sulfonylureas monotherapy.

We conducted a multicenter, randomized controlled trial to compare the effect of glimepiride and sitagliptin on the insulinogenic index after 52-week treatment in Japanese patients with T2DM.

## Subjects and methods

### Trial design and participants

A randomized, open-label, parallel-group trial was conducted over a period of 52 weeks from February 10, 2011 to March 31, 2013 at 18 centers across Japan, including clinics and general and university hospitals. Eligibility criteria were outpatients with T2DM aged < 80 years with an HbA1c level < 8.4 % who had received no pharmacological treatment for diabetes for at least 1 month prior to participation in this trial. Exclusion criteria were renal or liver dysfunction, pancreatic or hematological operation, severe complications of diabetes, being pregnant or possibly pregnant, malignancy under treatment and medications known to affect glucose metabolism.

### Ethics

The protocol was approved by the University hospital Medical Information Network (UMIN) (Clinical Trial Registry No. 000004791), the Ethics Committee of Kyoto University Graduate School and Faculty of Medicine, as well as the Ethics Committee of each study center. The trial was performed in accordance with the Declaration of Helsinki upon obtaining written informed consent from all participants, and was reported in accordance with the Consolidated Standards of Reporting Trials (CONSORT) Statement [26].

### Intervention and maintenance

Each participant was administered glimepiride (titrated upward to 1.0 mg) or sitagliptin (titrated upward to 100 mg) once daily in the morning for 52 weeks. The starting dose was decided by the respective physicians based on the baseline condition of each participant. When HbA1c levels exceeded 6.9 % after 6 months or later, glimepiride and sitagliptin doses were increased to each titrated dose. Physicians were allowed to decrease the doses at any point to prevent the occurrence of a hypoglycemic event. On the other hand, if participants did not meet the specified glycemic control criteria with the setup dose, physicians were allowed to add or switch medications and the participants were discontinued from the trial.

### Outcome measurements

The primary outcome measurement was the difference in post-treatment insulinogenic index between the two groups. Secondary outcome measurements were the levels of plasma glucose (PG) (mmol/l), immunoreactive insulin (IRI) (pmol/l), C-peptide (CPR) (nmol/l), glucagon (ng/l) (Millipore Corporation, Bilerica, MA), and insulin sensitivity index (ISI; an index of insulin resistance) during 75 g oral glucose tolerance tests (OGTTs) before and after 52-week treatment [27]. In addition, HbA1c (%), glycated albumin (GA) (%), and BMI (kg/m^2^) after treatment also were evaluated as secondary outcome measurements. Each outcome was calculated as follows: HbA1c was expressed as a National Glycohemoglobin Standardization Program (NGSP) equivalent value calculated by the following formula: HbA1c (NGSP value) (%) = 1.02 × HbA1c (Japan Diabetes Society value) (%) + 0.25 [28]. The estimated glomerular filtration rate (eGFR) (ml/min/1.73 m^2^) was calculated by 194 × serum Cr^−1.094^ × age^−0.287^ for men, 194 × serum Cr^−1.094^ × age^−0.287^ × 0.739 for women [29]. The insulinogenic index was calculated by the following equation: (IRI at 30 min - fasting IRI)/(PG at 30 min—fasting PG) [6–8]. ISI composite was calculated by 10,000/{[fasting PG (mmol/l) × fasting IRI (pmol/l) × mean 75 g OGTT PG (mmol/l) × mean 75 g OGTT IRI (pmol/l)]^0.5^}1/3 [27].

### Sample size

Given the lack of differences and variance in the insulinogenic index between two similar groups in a previous study, an effect size of 0.6, which is conventionally accepted as a medium effect, was used to calculate an appropriate sample size. We estimated that 100 participants would provide at least 80 % power to detect a statistically significant difference (α = 0.05, two-sided test, and withdrawal rate of 10 % per year) between the two groups.

### Randomization

We used the UMIN system, a computer-generated random sequence, to assign participants to either glimepiride or sitagliptin in a 1:1 ratio by minimization, based on sex, center, age, and HbA1c. Collaborating physicians enrolled the participants, and during the follow-up period, this trial was performed without blinding. That is, both physicians and participants were aware of which drug was allocated.

### Procedures

Upon obtaining informed consent, OGTTs were performed before (0 week) and after 52-week treatment. The levels of PG, IRI, CPR, and glucagon were measured at 0, 15, 30, 60, and 120 min (min) during OGTTs. After treatment, OGTTs were performed with a 24-h washout period. GA was measured at 0 and 52 weeks, and glutamic acid decarboxylase (GAD) antibody was measured at 0 week. HbA1c, PG, body weight, and clinical biochemical tests were measured (0, 4, 12, 24, 36, and 52 weeks). Safety monitoring for hypoglycemia was performed during treatment. All samples were labeled with a code assigned to each participant and routinely analyzed at a laboratory of the SRL Corporation (Tokyo, Japan).

### Statistical analyses

All statistical analyses were performed with a blind procedure by an independent third party, Statcom Company Limited. For the primary outcome measurement, the main analysis was performed on the full analysis set (FAS) of all randomized participants who took medications as allocated and underwent the OGTTs before and after treatment, excluding those with a hemolyzed sample, those who were added or changed therapy, and those who were withdrawn from the trial before treatment following consent acquisition. Subgroup analysis was performed on the per-protocol set (PPS), which excluded positive result of GAD antibody, protocol violations, or poor compliance from the FAS. Analysis of covariance (ANCOVA) was used to evaluate the primary outcome measurement based on baseline log-transformed insulinogenic index, and allocation variables, including age, sex, and HbA1c as covariates. For secondary outcome measurements, repeated measures analysis using a mixed model with terms for visit, treatment, and interaction was performed for OGTT, HbA1c, and BMI, including baseline values as covariates. Least-squares means (lsmeans) with 95 % confidence intervals (CIs) were obtained from the model, estimating from the mixed model. Other secondary outcome measurements were compared between the two groups using Analysis of variance (ANOVA) for the evaluation of GA and ISI. As an exploratory analysis, the change of insulinogenic index in each group was analyzed by Paired *t* test. The achievement rate of HbA1c < 7.0 % between the two groups were compared by Fisher’s Exact test. All statistical analyses were performed using SAS version 9.2 (SAS Institute Inc, Cary, NC) and JMP^®^ 9 (SAS Institute Inc., Cary, NC, USA). No interim analysis was performed.

## Results

### Flow chart of participants

**Fig. 1:**
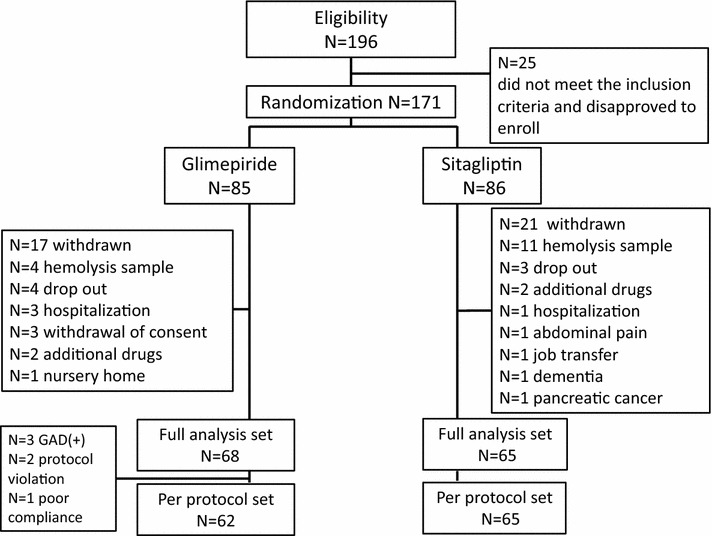
Flow chart of participation

A total of 196 participants were recruited for this trial (Fig. 1). Of these, 25 participants who did not meet the inclusion criteria were excluded. The remaining 171 participants were randomly assigned to sitagliptin or glimepiride groups in a 1:1 ratio. Of these, 133 participants (glimepiride, n = 68; sitagliptin, n = 65) were analyzed as the FAS, with a final follow-up rate of 77.8 %. In the glimepiride group, 62 participants were regarded as the PPS, which excluded six participants due to positive results of GAD antibody (n = 3), protocol violations (n = 2), or poor compliance (n = 1). In the sitagliptin group, the number of participants in the FAS and PPS was the same. The main reason for discontinuation in both groups was due to hemolysis of samples. Other reasons included dropout from treatment, addition of other drugs due to hyperglycemia, or insulin therapy under hospitalization.

### Demographics and participant characteristics in the FAS

All variables were well balanced between the two groups in FAS, and also divided into the same balance in the baseline (Table 1). Participants were middle-aged, had a mean BMI of 24.4 (3.6) (kg/m^2^), had short duration of diabetes, and had no severe renal dysfunction. They had started treatment with oral hypoglycemic agents at an HbA1c of 7.4 (0.5) %. Participant characteristics (i.e., low BMI, low insulin secretion, and low insulin resistance) are comparable to those of Asian T2DM patients previously reported [3, 7–12, 17, 18, 22–25]. The usual starting dose of glimepiride was 0.5 mg/day; that of sitagliptin was 50 mg/day. Eighteen patients had taken diabetic medication before enrollment as follows; biguanide (n = 1), insulin (n = 1), glinides (n = 4), alpha glucosidase inhibitors (n = 4), and sulfonylureas (n = 8). They had not used these antidiabetic treatments for at least 3 months before enrollment. Especially, eight patients who took sulfonylureas were divided into the two groups equally.

**Table 1:** Participant characteristics in the full analysis set

Variables	Glimepiride (n = 68)	Sitagliptin (n = 65)
Male/female (number, %)	49/19 (72.1/27.9 %)	49/16 (75.4/24.6 %)
Age (year)	64 (8)	63 (9)
BMI (kg/m^2^)	24.7 (3.3)	24.1 (3.8)
Duration (year)	6.0 (5.1)	6.2 (6.0)
HbA1c (NGSP, %)	7.5 (0.5)	7.4 (0.5)
GA (%)	19.5 (2.8)	19.4 (2.7)
eGFR (ml/min/1.73 m^2^)	74.9 (14.0)	76.2 (17.1)
ISI (l^2^/mmol pmol)	16.2 (9.2, 24.0)	16.3 (10.2, 22.0)
Log transformed insulinogenic index (pmol/mmol)	2.6 (2.1, 3.3)	2.2 (1.9, 2.8)
Starting dose (mg/day)	0.25 (20.6 %)	25 (12.3 %)
	0.5 (77.9 %)	50 (87.7 %)
	1.0 (1.5 %)	100 (0.0 %)

Data are expressed as means (SD), median with interquartile range (IQR), number (%), or percent (%)

Data were analyzed ANOVA or Fisher’s Exact test

No significant differences were observed between the two groups

*BMI* body mass index, *HbA1c* hemoglobin A1c, *ISI* insulin sensitivity index, *GA* glycated albumin, *eGFR* estimated glomerular filtration rate

### Primary outcome measurement

**Fig. 2:**
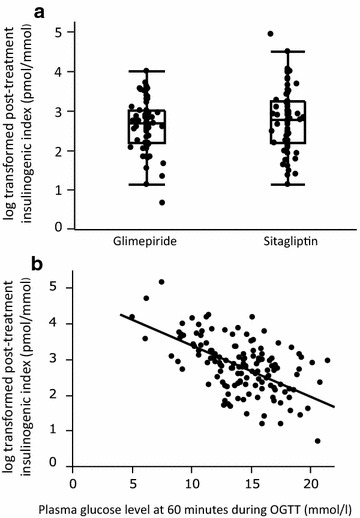
**a** *Box* and *dot plots* of log-transformed post-treatment insulinogenic index for glimepiride and sitagliptin groups. Analysis of covariance (ANCOVA) revealed a significantly higher post-treatment insulinogenic index in the sitagliptin group (p = 0.036) in the FAS. **b** Scatter plot and linear regression equation in total: log post-treatment insulinogenic index (pmol/mmol) = 4.8 − 0.1 × PG at 60 min (mmol/l) (R^2^, coefficient of determination = 35 %, p < 0.0001)

Insulinogenic index after 52-week treatment was significantly higher in the sitagliptin group than in the glimepiride group (p = 0.036) in the FAS (Fig. 2a). No interactions between the drugs and other adjusted factors were observed. Associations between insulinogenic indices and PG levels at 60, and 120 min during OGTT were evaluated. Insulinogenic indices were more negatively correlated with PG levels at 60 min than those at 120 min (R^2^ = 16 %, data not shown). The obtained linear regression equation in total is as follows: log post-treatment insulinogenic indices (pmol/mmol) = 4.8 − 0.1 × PG levels at 60 min (mmol/l) (R^2^, coefficient of determination = 35 %, p < 0.0001 in total, 38 %, 30 % in sitagliptin and in glimepiride, respectively) (Fig. 1b).

### Secondary outcome measurements

**Fig. 3:**
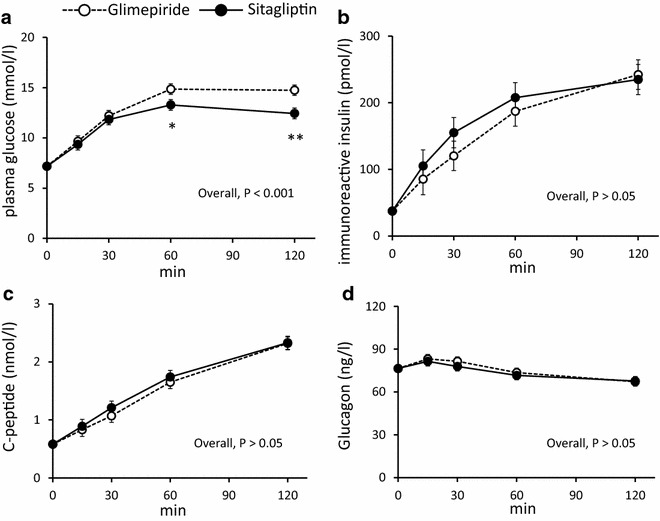
The levels of PG (**a**), IRI (**b**), CPR (**c**), and glucagon (**d**) during OGTT after 52-week treatment. *Filled circles* with a *dotted line*: post-glimepiride treatment; *filled circles* with a *solid line*: post-sitagliptin treatment. Values show least-squares mean with 95 % confidence interval (CI) estimated by a mixed-model for repeated measures analysis. **a** p < 0.001 glimepiride vs. sitagliptin, *p < 0.01, ** p < 0.001 vs. glimepiride at each time point. **b**–**d** not significant (n.s.) at each time point

The levels of PG, IRI, CPR, and glucagon during OGTTs were compared after both treatments (Fig. 3). PG levels at 60 min (p < 0.01) and 120 min (p < 0.001), and overall (p < 0.001) were significantly lower in the sitagliptin group than those in the glimepiride group (Fig. 3a). The levels of IRI, CPR, and glucagon did not differ between the two groups (Fig. 3b–d). We also compared the levels of PG, IRI, CPR, and glucagon during OGTTs between pre-treatment and post-treatment in each group (Additional file 1: Figure S1A–H). PG levels at 30, 60, and 120 min in glimepiride group were significantly lower after treatment than those before treatment (Additional file 1: Figure S1A), while PG levels at 60 and 120 min in sitagliptin group were significantly lower after treatment than those before treatment (Additional file 1: Figure S1B). In both groups, the level of overall glucose was significantly lower than that before treatment. The overall insulin level was significantly higher after treatment only in sitagliptin group (p < 0.05) (Additional file 1: Figure S1C, D). The level of overall CPR was increased after treatment in both groups compared to that before treatment (Additional file 1: Figure S1E, F) (p = 0.001, p < 0.001, respectively). All points of insulin and C-peptide levels during OGTT did not change between before and after 52 weeks treatment in each group. The level of glucagon, including overall level, was not significantly changed between before and after treatment in each group (Additional file 1: Figure S1G, H). Insulinogenic index after treatment was significantly higher than that before treatment in each group (p < 0.05, p < 0.001, respectively) (Data not shown).

HbA1c improved gradually from 7.4 % to 6.8 (6.7–7.0) % at 12 weeks, and remained the same until 52 weeks (Additional file 2: Figure S2). There was no significant difference in HbA1c levels between the two groups during 52-week treatment. Neither the post-treatment levels of HbA1c nor GA showed significant difference in the two groups (Table 2) (p = 0.79, p = 0.3, respectively). The achievement rate of HbA1c < 7.0 % also showed no significant difference between the two groups (61.8, 67.7 %, respectively, p = 0.586). ISI was significantly and slightly higher in sitagliptin group than that in glimepride group (p = 0.046). BMI did not differ after treatment (p = 0.75) (Table 2) and during the follow-up period (data not shown) between the two groups. At 52-week, the final dose of glimepiride was 0.25 mg/day (13.2 %), 0.5 mg/day (72.1 %), and 1.0 mg/day (14.7 %), and that of sitagliptin was 25 mg/day (6.2 %), 50 mg/day (83.1 %), 75 mg/day (1.5 %), and 100 mg/day (9.2 %).

**Table 2:** Post-treatment comparison of physical and chemical parameters in the full analysis set

Covariates	Glimepiride (n = 68)	Sitagliptin (n = 65)	p value^*^
BMI (kg/m^2^)	24.5 (24.2, 24.7)	24.5 (24.3, 24.8)	0.75
HbA1c (NGSP %)	6.8 (6.7, 7.0)	6.8 (6.7, 7.0)	0.79
GA (%)	17.4 (2.8)	17.7 (2.6)	0.30
ISI (l^2^/mmol pmol)	15.8 (9.4, 20.8)	17.3 (11.9, 23.5)	0.046^*^

Data are expressed as least-squares mean with 95 % confidence interval (CI) in BMI and HbA1c, means (SD) in GA, or median with interquartile range (IQR) in ISI

Analysis of variance (ANOVA) revealed a significant difference in the two groups

*BMI* body mass index, *HbA1c* hemoglobin A1c, *GA* glycated albumin, *ISI* insulin sensitivity index

^*^Comparison of the values after 52-week treatment between glimepiride and sitagliptin groups

The single, self-reported episode of mild hypoglycemia was experienced during exercise in the glimepiride group; no severe hypoglycemia was reported in either group. One participant in the sitagliptin group had abdominal pain and discontinued treatment. Three participants in the sitagliptin group showed more than a 1.3-fold increase in creatinine relative to baseline. Hepatic-related side effects were considered when laboratory values exceeded threefold upper limit of the normal range. Alanine aminotransferase (ALT) was elevated in five participants (glimepiride group, n = 1; sitagliptin group, n = 4); however, all had baseline ALT exceeding the limit of normal range. Aspartate aminotransferase (AST) was elevated in one participant in the glimepiride group.

## Discussion

In this trial, sitagliptin monotherapy resulted in significantly higher insulinogenic index compared with that of glimepiride monotherapy in Japanese patients with T2DM after 52-week treatment (Fig. 2a). It is reported that the DPP-4. Inhibitors conserved β-cell function in patients with T2DM and autoimmune diabetes [30, 31]. Our result is possibly due to conserving β-cell function by DPP-4 inhibitor. In addition, the insulinogenic index was negatively correlated with glucose levels at 60 min during OGTT after treatment (Fig. 2b). This result suggests that maintenance of the insulingenic index is important to preserve postprandial glucose levels in early T2DM and indicates that sitagliptin may have a better effect on lowering the postprandial plasma glucose levels than glimepride in Asian patients with T2DM. It was reported that DPP-4 inhibitors including sitagliptin suppress glucagon secretion [32, 33]. However, there were no effects of sitagliptin on glucagon secretion in this trial. This result suggests that the effect of sitagliptin on insulin secretion but not on glucagon secretion might continue after a drug washout period of 24 h after 52-week treatment.

Insulin secretion stimulated by sulfonylureas is independent of the glucose concentration, while DPP-4 inhibitors increase an active form of incretin peptide by DPP-4 inhibition and potentiate glucose-dependent insulin secretion [34–36]. Accordingly, sulfonylureas are associated with risk of hypoglycemia and weight gain; DPP-4 inhibitors are associated with lower frequency of hypoglycemia and are weight neutral [34–36]. In this trial, there was no hypoglycemia or weight gain in the sitagliptin group (Tables 1 and 2). However, it should be noted that glimepiride treatment also did not induce weight gain or incidence of severe hypoglycemia (Tables 1 and 2). This might be attributed to the low-dose of glimepiride used. Based on the Japanese claims database, 72 % of patients with single sulfonylurea treatment used 1.0 mg/day for 1 year, and 70 % of the patients treated with 1.0 mg/day of glimepiride achieved 1.0 % reduction of HbA1c [37]. Thus, the dose of glimepiride was set to 1.0 mg/day in this trial. Consequently, a similar reduction in HbA1c levels was observed during 52-week treatment and no significant differences were found in HbA1c and GA levels between the two groups (Table 2, Additional file 2: Figure S2). These results made it possible to evaluate insulin secretion under the same glucose control conditions. Accordingly, the dose selection of less than 1.0 mg/day of glimepiride, the effect of which on HbA1c reduction is comparable to that of less than 100 mg/day of sitagliptin, seemed reasonable in this trial. In addition, our results also suggest that in regard to HbA1c-lowering efficacy, a low-dose of glimepiride is similarly effective as sitagliptin without weight gain or severe hypoglycemia at the early stage of T2DM with low insulin secretion (Table 2 and Additional file 2: Figure S2). Although a similar reduction in HbA1c and GA was achieved, post-challenge plasma glucose levels were significantly lower with sitagliptin than with glimepiride (Table 2, Fig. 3a, and Additional file 2: Figure S2). This result indicates that sitagliptin has better effects on insulinogenic index after 24-h wash out period at 52-week treatment.

This trial has several limitations. First, low-dose glimepiride was used in Japanese patients with T2DM who have low BMI and insulin secretion [3, 7–9, 12, 17, 22–25]. The maximum dose of glimepiride was set at 1.0 mg/day to achieve the same improvement of HbA1c between the two groups. Although a significantly better level of insulinogenic index was shown by glimepiride treatment (p < 0.05, data not shown), it is unknown to what extent high-dose glimepiride improves the insulinogenic index or HbA1c in Japanese patients with T2DM. Second, meal tolerance tests (MTTs) were not performed in this trial. The primary endpoint is to evaluate early-phase insulin secretion in response to glucose. The insulinogenic index has already been established as an index of early-phase insulin secretion during OGTT [6–8]. However, the indices of the meal-stimulated insulin secretion by MMTs have not established, mainly because the total calories and contents of the meal differ among the previous studies [32, 33]. Third, the follow up rate of 78 % might be relatively low. The main reason is hemolysis of samples, which are important for the calculation of insulinogenic index. If we had planned to use multiple imputation method for missing data in the protocol, we might obtain higher follow up rate. The strength of this trial lies in its design, i.e., a multicenter, randomized, controlled trial involving clinics and a university hospital, which enhances the generalizability of our results. Furthermore, an active-controlled trial comparing the most widely used insulin secretagogues is practical for a daily clinical setting. This trial focused on the pathophysiology and treatment efficacy of T2DM in Asia. Our finding of a better effect on early-phase insulin secretion is of clinical importance for Asian patients with T2DM.

In conclusion, sitagliptin showed better effects on insulinogenic index after 52-week treatment compared to glimepiride in Japanese patients with T2DM. Further research is required to assess early-phase insulin secretion in patients treated with these drugs for longer period.

## Additional files

10.1186/s13098-016-0131-y The levels of PG (A and B), IRI (B and D), CPR (E and F), and glucagon (G and H) during OGTT before and after 52-week treatment in glimepiride (A, C, E, and G) and sitagliptin groups (B, D, F, and H). Outlined circles with a dotted line: pre-glimepiride treatment, filled circles with a solid line: post-glimepiride treatment. Outlined squares with a dotted line: pre-sitagliptin treatment, filled squares with a solid line: post-sitagliptin treatment. Values show least-squares mean with 95 % confidence interval (CI) estimated by a mixed-model for repeated measures analysis. Asterisks indicate significant differences between pre-treatment and post-treatment at each time point († p < 0.05, †† p < 0.01, ††† p < 0.001). A) p < 0.001 pre- vs. post-glimepiride. † p < 0.05 at 30 and 60 min and †† p < 0.01 at 120 min pre- vs. post-glimepiride. B) p < 0.001 pre- vs. post-sitagliptin. ††† p < 0.001 at 60 and 120 min pre- vs. post-sitagliptin, C) not significant (n.s.) at each time point, D) p < 0.05 pre- vs. post-sitagliptin, E) p = 0.001 pre- vs. post-glimepiride, F) p < 0.001 pre- vs. post-sitagliptin, G) n.s. at each time point, H) n.s. at each time point.
10.1186/s13098-016-0131-y Time course of HbA1c from baseline to 52 weeks in the FAS. White circles with a dotted line: glimepiride group, filled black circles with a solid line: sitagliptin group. Values show least-squares mean with 95 % confidence interval (CI) estimated by a mixed-model for repeated measures analysis, including terms for baseline HbA1c visit and treatment by visit interaction. There was no significant difference between the two groups at any point.

## Abbreviations

T2DM: type 2 diabetes mellitus
BMI: body mass index
DPP-4: dipeptidyl peptidase-4
HbA1c: hemoglobin A1c
PG: plasma glucose
IRI: immunoreactive insulin
CPR: C-peptide
ISI: insulin sensitivity index
OGTT: 75 g oral glucose tolerance tests
GA: glycated albumin
GAD: glutamic acid decarboxylase
FAS: full analysis set
PPS: per protocol set
ANCOVA: analysis of covariance
ANCOVA: analysis of covariance
CI: confidence intervals
SD: standard deviation
IQR: interquartile range
ALT: alanine aminotransferase
AST: aspartate aminotransferase
