# Supplementary figures and images for: Sitagliptin monotherapy has better effect on insulinogenic index than glimepiride monotherapy in Japanese patients with type 2 diabetes mellitus: a 52-week, multicenter, parallel-group randomized controlled trial
Source: Diabetol Metab Syndr. 2016 Feb 27;8:15. doi: 10.1186/s13098-016-0131-y (PMC4769515; doi:10.1186/s13098-016-0131-y)

FigureS1

## Glimepiride

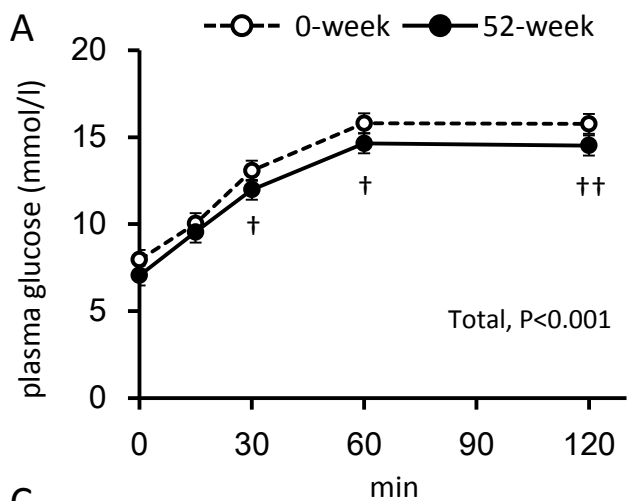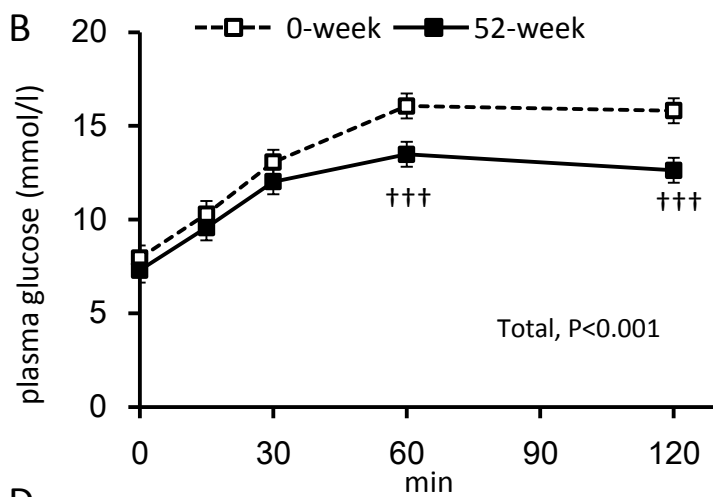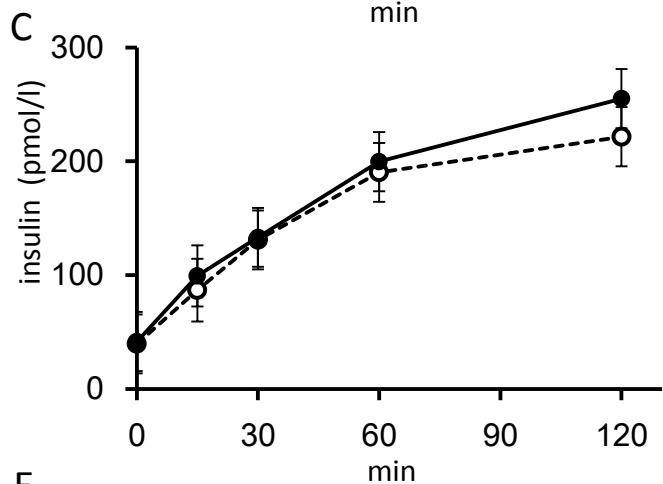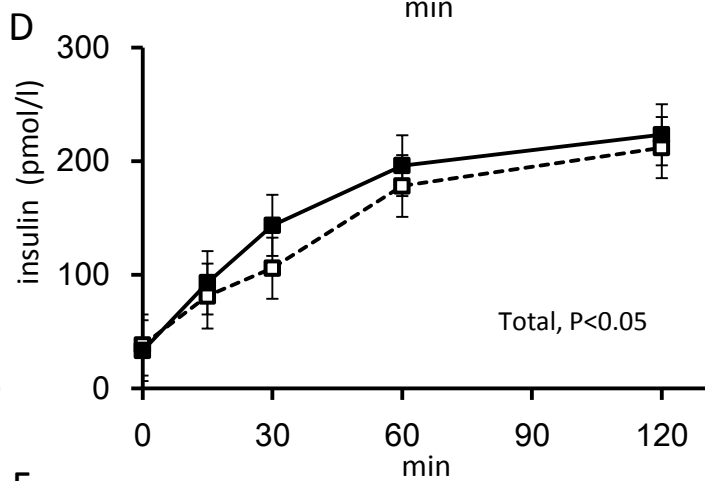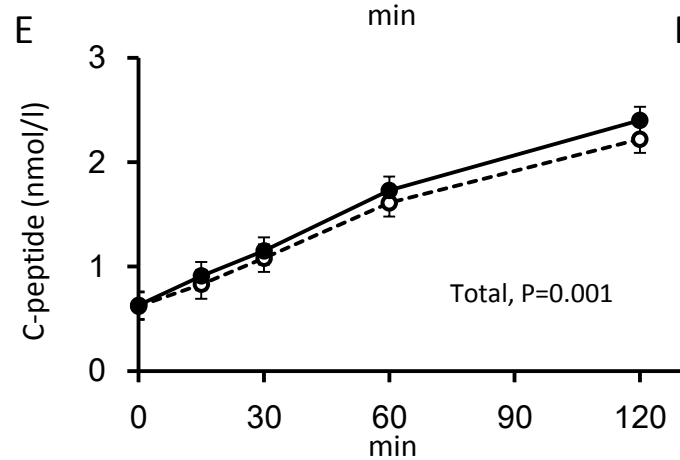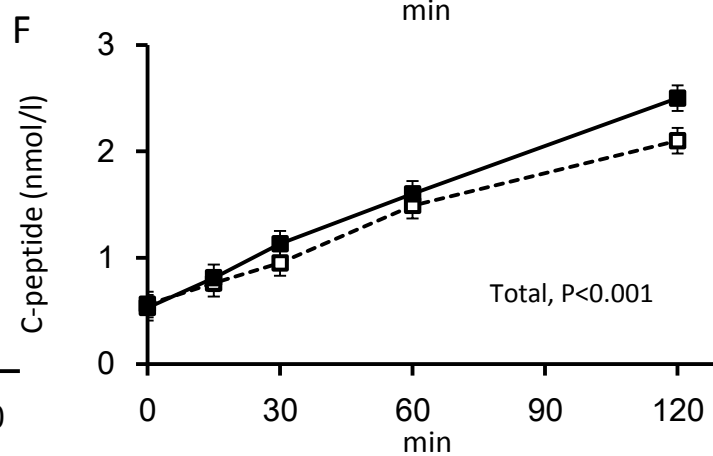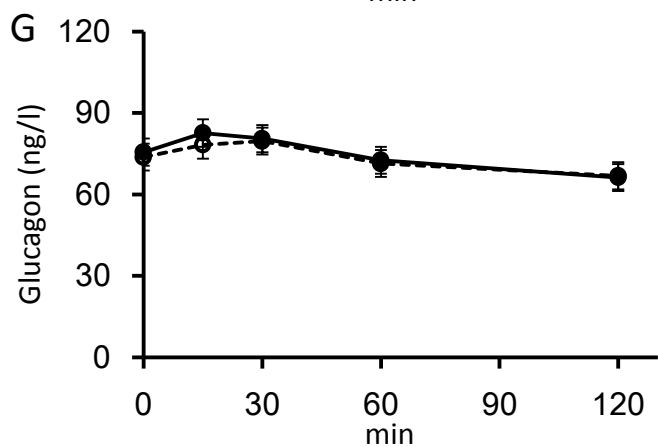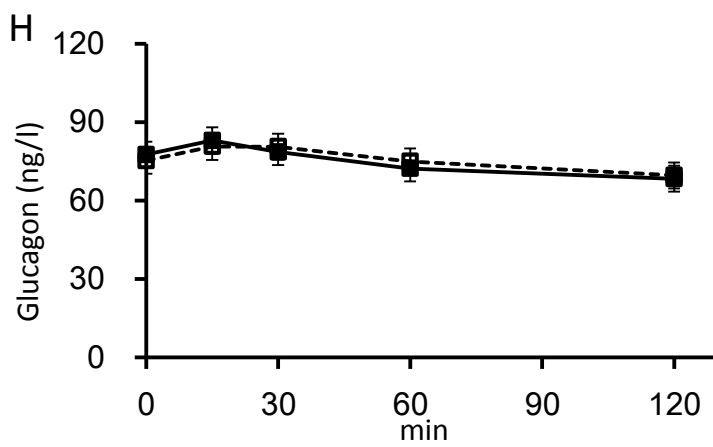

Supplement: Supplementary file 1 — 10.1186/s13098-016-0131-y The levels of PG (A and B), IRI (B and D), CPR (E and F), and glucagon (G and H) during OGTT before and after 52-week treatment in glimepiride (A, C, E, and G) and sitagliptin groups (B, D, F, and H). Outlined circles with a dotted line: pre-glimepiride treatment, filled circles with a solid line: post-glimepiride treatment. Outlined squares with a dotted line: pre-sitagliptin treatment, filled squares with a solid line: post-sitagliptin treatment. Values show least-squares mean with 95 % confidence interval (CI) estimated by a mixed-model for repeated measures analysis. Asterisks indicate significant differences between pre-treatment and post-treatment at each time point († p < 0.05, †† p < 0.01, ††† p < 0.001). A) p < 0.001 pre- vs. post-glimepiride. † p < 0.05 at 30 and 60 min and †† p < 0.01 at 120 min pre- vs. post-glimepiride. B) p < 0.001 pre- vs. post-sitagliptin. ††† p < 0.001 at 60 and 120 min pre- vs. post-sitagliptin, C) not significant (n.s.) at each time point, D) p < 0.05 pre- vs. post-sitagliptin, E) p = 0.001 pre- vs. post-glimepiride, F) p < 0.001 pre- vs. post-sitagliptin, G) n.s. at each time point, H) n.s. at each time point. [file 13098_2016_131_MOESM1_ESM.pdf]

FigureS2

NGSP (%)

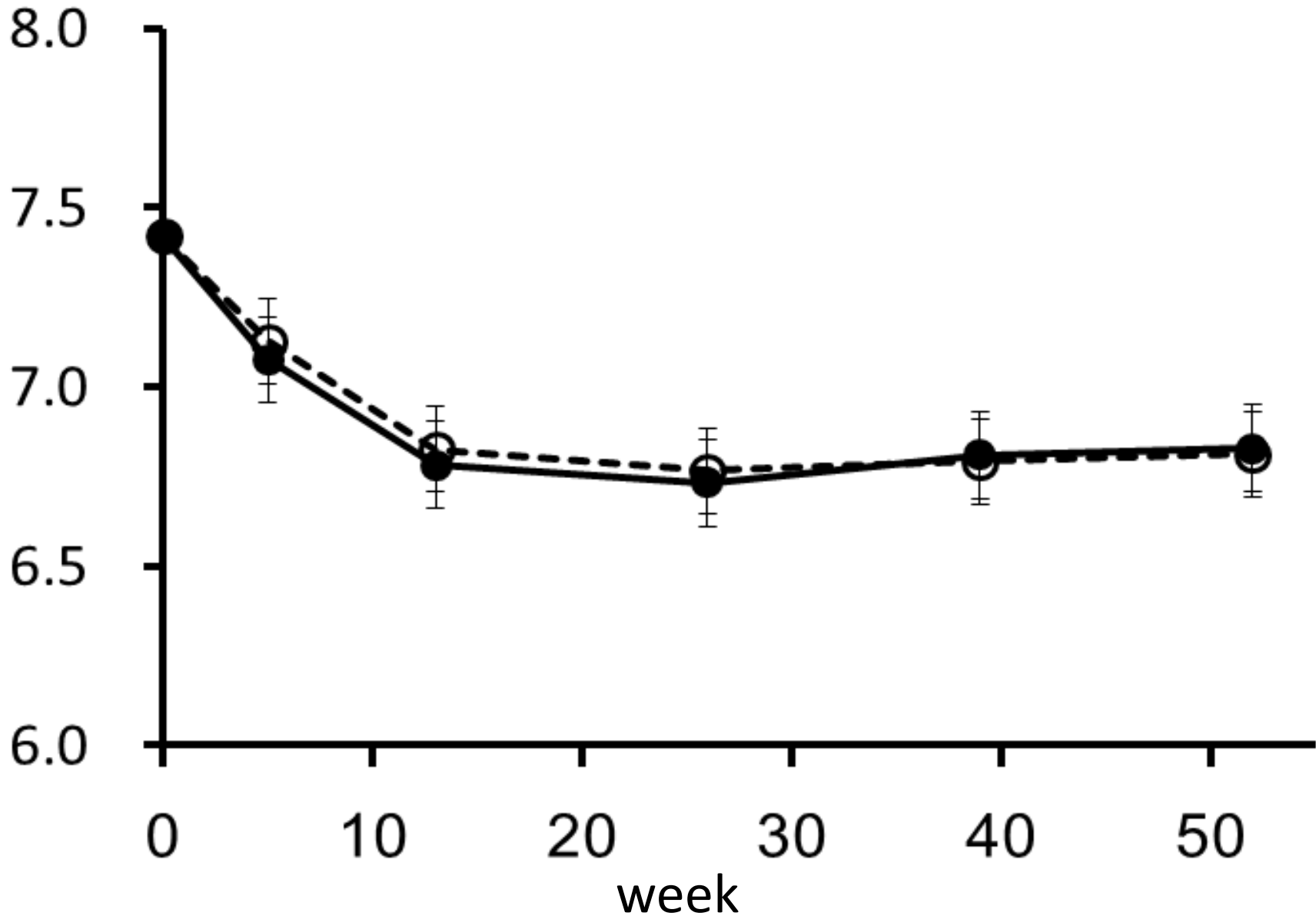

Supplement: Supplementary file 2 — 10.1186/s13098-016-0131-y Time course of HbA1c from baseline to 52 weeks in the FAS. White circles with a dotted line: glimepiride group, filled black circles with a solid line: sitagliptin group. Values show least-squares mean with 95 % confidence interval (CI) estimated by a mixed-model for repeated measures analysis, including terms for baseline HbA1c visit and treatment by visit interaction. There was no significant difference between the two groups at any point. [file 13098_2016_131_MOESM2_ESM.pdf]
